# Direct notification of cervical cytology results to women improves follow-up in cervical cancer screening - A cluster-randomised trial

**DOI:** 10.1016/j.pmedr.2018.11.015

**Published:** 2018-11-23

**Authors:** Bettina Kjær Kristiansen, Berit Andersen, Flemming Bro, Hans Svanholm, Peter Vedsted

**Affiliations:** a Research Unit for General Practice, Department of Public Health, Aarhus University, 8000 Aarhus, Denmark; b Department for Public Health Programmes, Randers Regional Hospital, 8930 Randers, Denmark; c Department of Pathology, Randers Regional Hospital, 8930 Randers, Denmark; d Research Centre for Cancer Diagnosis in Primary Care (CaP), Department of Public Health, Aarhus University, 8000 Aarhus, Denmark

**Keywords:** AGC, Atypical Glandular Cells, AIS, adenocarcinoma in situ, ASC-H, atypical squamous cells cannot exclude HSIL, ASC-US, Atypical Squamous Cells of Undetermined Significance, CCU, cancer of the cervix uteri, CDR, Central Denmark Region, DPDB, Danish National Pathology Registry and Data Bank, GP, general practitioner, hrHPV-pos., high-risk Human Papilloma Virus positive, ICC, intra-cluster correlation coefficient, HSIL, High-grade Squamous Intraepithelial Lesion, LSIL, Low-grade Squamous Intraepithelial Lesion, PD, prevalence differences, PR, prevalence ratio, SNOMED, Systematized Nomenclature of Medicine, General practice, Uterine cervical dysplasia, Mass screening, Early detection of cancer, Socioeconomic factors, Quality of health care

## Abstract

Up to half of all women do not receive follow-up as recommended after cervical cytology testing and are thus at increased risk of dysplasia progression. Women from lower social positions are at increased risk of not receiving follow-up. Sample takers, often general practitioners, convey results to women, but communication problems constitute a challenge. We aimed to investigate the effect of direct notification of cervical cytology results on follow-up rates.

In a 1:1 cluster-randomised controlled trial, we assessed if having the pathology department convey cervical cytology results directly to the investigated women improved timely follow-up, compared with conveying the results via the general practitioner as usual. All women with a cervical cytology performed in a general practice in the Central Denmark Region (2013–2014) and receiving follow-up recommendation were included (*n* = 11,833).

The proportion of women without timely follow-up was lower in the group with direct notifications than in the control group of women receiving usual care, regardless of age, educational status, cohabitation status and ethnicity. Among the women with the most severe cervical cytology diagnoses who are recommended gynaecological follow-up within 3 months, the percentage without timely follow-up was 15.1% in the intervention group and 19.5% in the control group (prevalence difference: −0.04 (95%CI: −0.07; −0.02)). Improved timely follow-up was also observed for women with a recommendation to have follow-up performed at 3 and 12 months.

Cervical cytology results conveyed directly by letter to women increased the proportion of women with timely follow-up without raising inequality in follow-up measured by social position.

**Trial registration**: ClinicalTrials.gov (TRN: NCT02002468) 29 November 2013.

## 1 Introduction

It is widely recognised that implementation of cervical cancer screening reduces the incidence and mortality associated with cervical cancer (Arbyn et al., 2008). Even so, cancer of the cervix uteri (CCU) remains the third most common cancer among women under 50 years of age in the Scandinavian countries (Engholm et al., n.d.). Reasons for stagnation of the decline in CCU incidence, may be explained by poor coverage and missed, delayed or insufficient follow-up on abnormal cervical cytology (Lynge et al., 2017). Women participating in CCU screening and having an abnormal cervical cytology but no follow-up do not benefit from participating in the programme. The proportion of women who are in need of but do not attend follow-up ranges from 7 to 49% depending on the setting (Yabroff et al., 2000; Styregruppen for DKLS, n.d.-a; Kupets et al., 2014). A meta-analysis suggests that 11.9% (95% CI: 9.0–15.6) of CCU cases in countries with organised screening programmes are due to missed follow-up of abnormal test results (Spence et al., 2007).

Missed follow-up has been associated with organisational and logistic deficiencies among sample takers (Yabroff et al., 2000; Yabroff et al., 2011; Bowie et al., 2014). In many countries, general practitioners (GPs) perform the vast majority of the cervical cytology testing and also convey the results to the women, and are responsible for the follow-up process (Arbyn et al., 2008; Anttila et al., 2009; Elfstrom et al., 2015; Linos and Riza, 2000). Several studies suggest that communication of cervical cytology results to women may be delayed, not conveyed, or misunderstood, which may lead to loss of recommended follow-up (Arbyn et al., 2008; Bowie et al., 2014; Philips et al., 2002; Bro et al., 2008; Simon et al., 2010). Furthermore, missed follow-up has been associated with a lower social position among women (Elit et al., 2012; Lindau et al., 2006; Hui et al., 2014; Eggleston et al., 2007; Schofield et al., 1994) and may lead to sociodemographic inequalities with respect to cervical cancer stage and survival (Ibfelt et al., 2012; Ibfelt et al., 2013). This calls for initiatives to decrease delay in cervical cancer diagnosing, without reinforcing existing disparities (Engholm et al., n.d.; Ibfelt et al., 2012; Lynge et al., 2014). It has been hypothesised that direct communication of cervical cytology results from the pathology departments to the tested woman may improve follow-up (Sundhedsstyrelsen, 2012). We therefore conducted a cluster-randomised controlled trial to evaluate the effect of direct notification of cervical cytology results on the proportion of women without timely follow-up.

## 2 Methods

### 2.1 Setting

The study was carried out in the Central Denmark Region (CDR) which is one of five Danish regions. The CDR covers approx. 22% of the Danish population including 340,000 women in the target population for CCU screening (Statistics Denmark, n.d.) and 420 general practices with one or more GPs. In total, 99% of all Danish citizens are listed with a specific GP. Cervical cytologies can be taken after invitation or opportunistically, or as part of a follow-up programme. Approximately 85% of samples in CDR are obtained in general practices. All services regarding screening and follow-up are free of charge for the patient, as GPs are reimbursed by the Danish health authorities (Vedsted et al., 2005). Danish women are invited by postal letter by the authorities to participate in regular cervical cancer screening; women aged 23–49 years are invited every third year, and women aged 50–64 years are invited every fifth year. Women should contact their GP to have a cytology taken. National standards from 2007 and 2012 describe how pathology departments using specific codes from the Danish version of the Systematized Nomenclature of Medicine (SNOMED) must classify diagnoses according to the 2001 Bethesda System and apply a follow-up recommendation if needed (Sundhedsstyrelsen, 2012; Sundhedsstyrelsen, 2007; Solomon et al., 2002). More than 10% of cervical cytology results require follow-up. In these cases results may either be abnormal, inadequate or normal (as part of a surveillance programme due to earlier abnormalities). In 2012, 20% of the Danish women with abnormal or inadequate results had not attended follow-up 3 months after the recommended follow-up date (Styregruppen for DKLS, n.d.-b). In these cases, the screening programme automatically generates a reminder which is sent to the sample taker. If the sample taker is a GP, the reminder is sent to the inbox of the general practice mailing system (Styregruppen for DKLS, n.d.-c). In Denmark all sample takers are legally responsible for securing follow-up.

### 2.2 Design

In a cluster-randomised trial, all general practices in the CDR were allocated to either an intervention group or a control group.

### 2.3 Intervention

All women listed with intervention general practices received a direct notification of the cytology result by postal letter. An SNOMED-code algorithm determined which combinations of SNOMED-codes that were allowed for a woman to receive a specific type of result notification (see Appendix A). The woman's current address was obtained from the Danish Civil Registration System, and dates and diagnostic information on the cervical cytology were retrieved from the Danish National Pathology Registry and Data Bank (DPDB) (Erichsen et al., 2010). The notifications were written in lay language (Kavanagh and Broom, 1997) and described the cervical cytology result as either “normal”, “abnormal” or “inadequate”, as recommended by the Danish Health Authority (Sundhedsstyrelsen, 2012). This description was accompanied by information on regular screening intervals or by a follow-up recommendation. The follow-up recommendations were divided into four groups; within 3 months (severe cervical cytology result prompting referral to a gynaecologist) or in 3, 6 or 12 months. The algorithm was pilot-tested during a 3-month period (10 October 2012–6 January 2013). A final algorithm was implemented on 7 January 2013. Intervention GP practices received results electronically from pathology departments, as always.

### 2.4 Control

Women listed with control general practices received no direct notification, but their GPs received their results electronically from pathology departments and conveyed results to women by phone, e-mail or at a face-to-face consultation, as usual.

### 2.5 Participants

Women aged 23–64 years were eligible for inclusion in this study if they had a cervical cytology performed in a general practice in the period from 7 January 2013 to 1 June 2014 and were recommended follow-up. Exclusion criteria were similar for both randomisations groups. We excluded cervical cytologies with follow-up recommendations for the following reasons: 1) Unknown postal address in the Danish Civil Registration System, as they could not receive a direct notification. In these situations, intervention GPs were informed by postal letter to convey results to women. 2) Timing of relevant follow-up was unclear. In these situations, the direct notification letter advised intervention women to contact their GP to have the result conveyed. 3) Women who died or emigrated from Denmark in the observation period. 4) Finally, in case of more than one cytology, only the first cytology performed in each woman was included.

### 2.6 Data

The DPDB is updated several times per day with cervical cytology results, and at the same time an electronically result is sent to the GP. Cervical cytology results from the DPDB were used to generate direct results notifications three times per week in the study period.

Data on histology or cytology follow-up were retrieved from the DPDB on 1 January 2016.

Sociodemographic characteristics on 1 January in the year preceding the cervical cytology were obtained from Statistics Denmark for educational attainment, cohabitation status and ethnicity. Educational attainment was classified as low (≤10 years), medium (>10 ≤ 15 years) or high education (>15 years) (International Standard Classification of Education, 2014). Cohabitation status was categorised as married/cohabitating or living alone. Ethnicity was categorised as Danish, immigrant/descendant from a Western country or immigrant/descendant from a non-western country (Statistics Denmark, n.d.). Age was calculated at the date of the cytology requisition and categorised as 23–34, 35–44, 45–54 or 55–64 years of age. The unique civil registration number (CRN) assigned to all Danes at birth allowed for individual level linkage of registry data (Schmidt et al., 2014).

### 2.7 Statistical analysis

The proportion of women without follow-up was estimated as prevalence proportions for each randomisation group. “No follow-up” was defined as absence of a new cervical cytological or histological sample (i.e. including biopsies) at different relevant time points. The results were presented as totals and stratified by type of follow-up recommendation using a binomial regression with logarithmic link function to accommodate the estimation of prevalence ratios (PRs) and prevalence differences (PDs). Kaplan-Meier functions were used to display the time from sample requisition to follow-up or censuring due to missed follow-up, whichever occurred first.

To determine if the effect of the intervention was modified by sociodemographic factors, proportions of women without follow-up were compared for different sociodemographic groups; both on a relative scale and on an absolute scale (Harper et al., 2010) with binomial regression, as described above.

All analyses were performed unadjusted and adjusted for educational status and numbers of GPs per cluster. Analyses were performed as intention-to-treat analyses, and robust standard errors were used to correct for clusters of GPs (Donner and Klar, 2000). The intra-cluster correlation coefficient (ICC) was determined by the STATA command “loneway”. All statistical analyses were conducted using STATA, version 14.

### 2.8 Sample size calculations

Calculations were based the smallest relevant subgroup. This was the 4.7% (95% CI: 3.8 to 5.8) of women with carcinoma, high-grade intraepithelial lesion (HSIL), adenocarcinoma in situ (AIS), atypical squamous cells cannot exclude HSIL (ASCH) and atypical glandular cell (AGC) cytologies who were not followed up timely in 2010 (Styregruppen for DKLS, n.d.-a). If a relevant reduction down to 2% for the intervention group was assumed, a total of 1394 women (power of 80% in a two-sided test with alpha level set at 5%) needed to be included. To allow for the design effect of clustering, the study period was pragmatically timed to ensure inclusion of twice that many women.

### 2.9 Randomisation and masking

Direct notification of cervical cytology results may influence how general practices organise their communication with the women. To allow intervention practices to adjust their routines, they were informed about the study. As cooperation between practices at the same address was likely, allocation was based on clusters of practices at similar addresses. A data manager without knowledge of the trial randomised the 340 GP clusters in STATA, version 12 in September 2012, before the pilot study.

### 2.10 Ethics

The Danish Data Protection Agency gave permission to use the registry data (ID 2009-41-3471 and ID 2012-41-0728/1-16-02-376-16). The Danish Health Authority gave permission to use the data from the medical records of the DPDB (ID 3-3013-1371/1). The Committee on Health Research Ethics in the CDR found that no ethical approval was required for this study (ID 211/2011). The study is registered with ClinicalTrials.gov (TRN: NCT02002468)*.*

## 3 Results

**Fig. 1:**
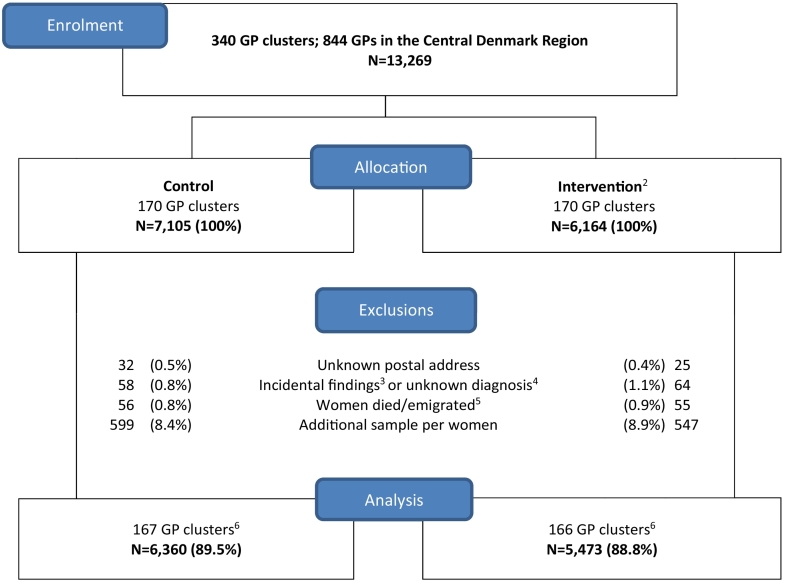
Flow chart for cervical cytologies^1^ obtained in general practice in the Central Denmark Region with a follow-up recommendation from 7 January 2013 to 1 June 2014 for women aged 23–64 years. Clusters consisted of general practices at the same address (*N* = number of cervical cytologies). ^1^Samples with topography SNOMED codes: T8X210, T8X310, T8X311, T8X312, T8X320, or T8X330. ^2^One intervention practice did not wish to participate; these women received usual care, but the analysis was performed as intention to treat. ^3^Other organisms or non-neoplastic findings, for example herpes. ^4^Ambiguous SNOMED coding, unprecise follow-up recommendation or uncommon topography (i.e. T8X210, T8X311, T8X312, T8X320, or T8X330). ^5^Observation period ended four months after GP reminder date. ^6^Seven general practices did not obtain cervical cytologies.

In total, 121,473 cervical cytologies were obtained in the CDR during approximately 17 months. The majority (108,204 (89.1%)) did not have a follow-up recommendation. Thus, 13,269 (10.9%) cytologies were eligible for inclusion and 11,833 of these were included in the analyses (Fig. 1).

Table 1 shows that characteristics of included clusters and women were distributed similarly in the intervention and control groups, except for the number of GPs per cluster and the women's educational level.

**Table 1:** Baseline characteristics of women and their cervical cytology sample included in the study. Clusters consisted of cluster-randomised general practitioners (GPs) in practices at the same address.

	Control	Intervention	*P*-values^c^
GP clusters^a^	167 (100%)	166 (100%)	
Number of GPs per GP cluster			
1 GP	45 (26.9%)	55 (33.1%)	
2–4 GPs	107 (64.1%)	94 (56.6%)	
>5 GPs	15 (9.0%)	17 (10.2%)	0.38
GP clusters without male GPs	22 (13.2%)	22 (13.3%)	0.98
GP clusters without female GPs	58 (34.7%)	60 (36.1%)	0.78
Median number of samples per GP cluster (10th to 90th percentile)	24 (4 to 79)	21 (4 to 57)	0.12
Number of women/cervical cytology samples	6360 (100%)	5473 (100%)	
Diagnosis^b^			
Normal	1604 (25.2%)	1386 (25.3%)	
Inadequate	872 (13.7%)	772 (14.1%)	
hrHPV-pos. (high risk Human Papilloma Virus positive)	228 (3.6%)	160 (2.9%)	
ASC-US (Atypical Squamous Cells of Undetermined Significance)	1116 (17.6%)	1028 (18.8%)	
LSIL (Low-grade Squamous Intraepithelial Lesion)	1036 (16.3%)	874(16.0%)	
ASC-H (atypical squamous cells cannot exclude HSIL)	710 (11.2%)	596 (10.9%)	
AGC (Atypical Glandular Cells)	85 (1.3%)	71 (1.3%)	
HSIL (High-grade Squamous Intraepithelial Lesion), AIS (adenocarcinoma in situ)	696 (11.0%)	578 (10.6%)	
Carcinoma	13 (0.2%)	8 (0.2%)	0.42
Follow-up recommendation			
Within 3 months	2982 (46.9%)	2513 (45.9%)	
In 3 months	846 (13.3%)	751 (13.7%)	
In 6 months	89 (1.4%)	68 (1.2%)	
In 12 months	2443 (38.4%)	2141 (39.1%)	0.59
Age group (years)			
23–34	2977 (46.8%)	2528 (46.2%)	
35–44	1687 (26.5%)	1448 (26.5%)	
45–54	965 (15.2%)	829 (15.2%)	
55–64	731 (11.5%)	668 (12.2%)	0.68
Ethnicity			
Danish	5861 (92.2%)	5089 (93.0%)	
Western immigrants/descendants	168 (2.6%)	154 (2.8%)	
Non-western immigrants/descendants	283 (4.5%)	197 (3.6%)	
Missing	48 (0.8%)	33 (0.6%)	0.08
Cohabitation status			
Married/cohabitating	3802 (59.8%)	3300 (60.3%)	
Living alone	2510 (39.5%)	2140 (39.1%)	
Missing	48 (0.8%)	33 (0.6%)	0.54
Education (years)			
≤10	959 (15.8%)	931 (17.0%)	
>10 ≤ 15	3077 (48.4%)	2768 (52.6%)	
>15	2125 (33.4%)	1593 (29.1%)	
Missing	199 (3.1%)	181(3.3%)	0.00

a 340 GP-address clusters were randomised, but seven general practices did not obtain a cervical cytology test.

b Appendix B shows the distribution of diagnoses generating the follow-up recommendation.

c Chi^2^ test for categorical data, and *t*-test for continues data.

Ninety-five percent of cervical cytology results were sent to GPs within 10 days from the requisition date, and 90% of all direct notifications to women in the intervention group were dispatched within 14 days from the requisition date (results not shown). Few women experienced a longer dispatch delay, as notifications were not sent just before weekends or holidays, as the women would otherwise not have been able to get in touch with their GP upon the arrival of their direct notification.

Table 2 shows the proportion of women without follow-up in both randomisations groups by type of follow-up recommendation at different time points, with the latest time point being when a GP reminder was generated. For most types of follow-up recommendations, more women had received timely follow-up in the intervention group than in the control group. Adjustment for educational status and number of GPs per cluster only changed the estimate slightly (results not shown).

**Table 2:** The effect of direct notification of cervical cytology results to the women measured as prevalence ratio (PR) and prevalence differences (PD) of not having follow-up at different time points. Effects are presented in total and stratified by type of follow-up recommendation (i.e. recommended gynaecological follow-up, or follow-up in 3, 6 or 12 months).

	Control %	Intervention %	PR (95% CI)	PD (95% CI)
Total	*n* = 6360	*n* = 5473		
No timely follow-up	47.2	42.9	**0.91 (0.86; 0.96)**	**−0.04 (−0.07; −0.02)**
No follow-up one month after recommended follow-up	35.0	31.4	**0.90 (0.84; 0.95)**	**−0.04 (−0.06; −0.02)**
Recommended follow-up in 12 months^a^	*n* = 2443	*n* = 2141		
No timely follow-up	76.6	73.7	**0.96 (0.93; 1.00)**	**−0.03 (−0.05; −0.00)**
No follow-up one month after recommended follow-up	63.2	60.4	0.96 (0.91; 1.00)	**−**0.03 (−0.06; 0.02)
No follow-op three months after recommended follow-up	46.7	46.4	0.99 (0.91; 1.07)	−0.00 (−0.04; 0.03)
Recommended follow-up in 6 months	*n* = 89	*n* = 68		
No timely follow-up	65.2	52.9	0.81 (0.61; 1.07)	−0.12 (−0.28; 0.04)
No follow-up one month after recommended follow-up	47.2	38.2	0.81 (0.55; 1.19)	−0.09 (−0.25; 0.07)
No follow-op three months after recommended follow-up	25.8	26.5	1.02 (0.60; 1.76)	0.01 (−0.14; 0.15)
Recommended follow-up in 3 months^b^	*n* = 846	*n* = 751		
No timely follow-up	57.9	47.3	**0.81 (0.73; 0.92)**	**−0.11 (−0.17; −0.05)**
No follow-up one month after recommended follow-up	39.6	31.8	**0.80 (0.69; 0.94)**	**−0.08 (−0.13; −0.02)**
No follow-op three months after recommended follow-up	27.2	22.9	0.84 (0.70; 1.02)	−0.04 (−0.09; 0.01)
Recommended gynaecological follow-up within 3 months^c^	*n* = 2982	*n* = 2513		
No timely follow-up	19.5	15.1	**0.77 (0.66; 0.91)**	**−0.04 (−0.07; −0.02)**
No follow-up one month after recommended follow-up	10.2	6.4	**0.63 (0.52; 0.75)**	**−0.04 (−0.05; −0.02)**

Bold characters = p-value < 0.05

a 63% of the women in this group had a normal result but were recommended follow-up as surveillance of earlier abnormal results (See Appendix B for the distribution of cervical cytology results by follow-up recommendation).

b 99% of the women in this group had inadequate results (See Appendix B).

c 23% of the women in this group had AIS/HSIL, 23% had ASCH, and 29% had hrHPV-pos. ASCUS or LSIL (See Appendix B).

Stratifying women with a recommended gynaecological follow-up within 3 months, depending on severity of cervical cytology results, revealed a PR 1 month after recommended follow-up for women with ASCH and HSIL/AIS at 0.48 (0.28; 0.83) and 0.51 (0.27; 0.98), respectively. Women with less severe results had no significant effect of the intervention (i.e. hrHPV-pos. ASCUS at 0.56 (0.32; 1.01) and hrHPV-pos. LSIL at 0.74 (0.43; 1.25)).

Stratifying women with a recommended follow-up in 12 months into abnormal or normal cervical cytology results showed that women with abnormal results had an effect of the intervention 1 month after the date of recommended follow-up with a PR at 0.85 (0.76; 0.95), whereas women with normal results had no effect (i.e. 1.00 (0.96; 1.05)).

**Fig. 2:**
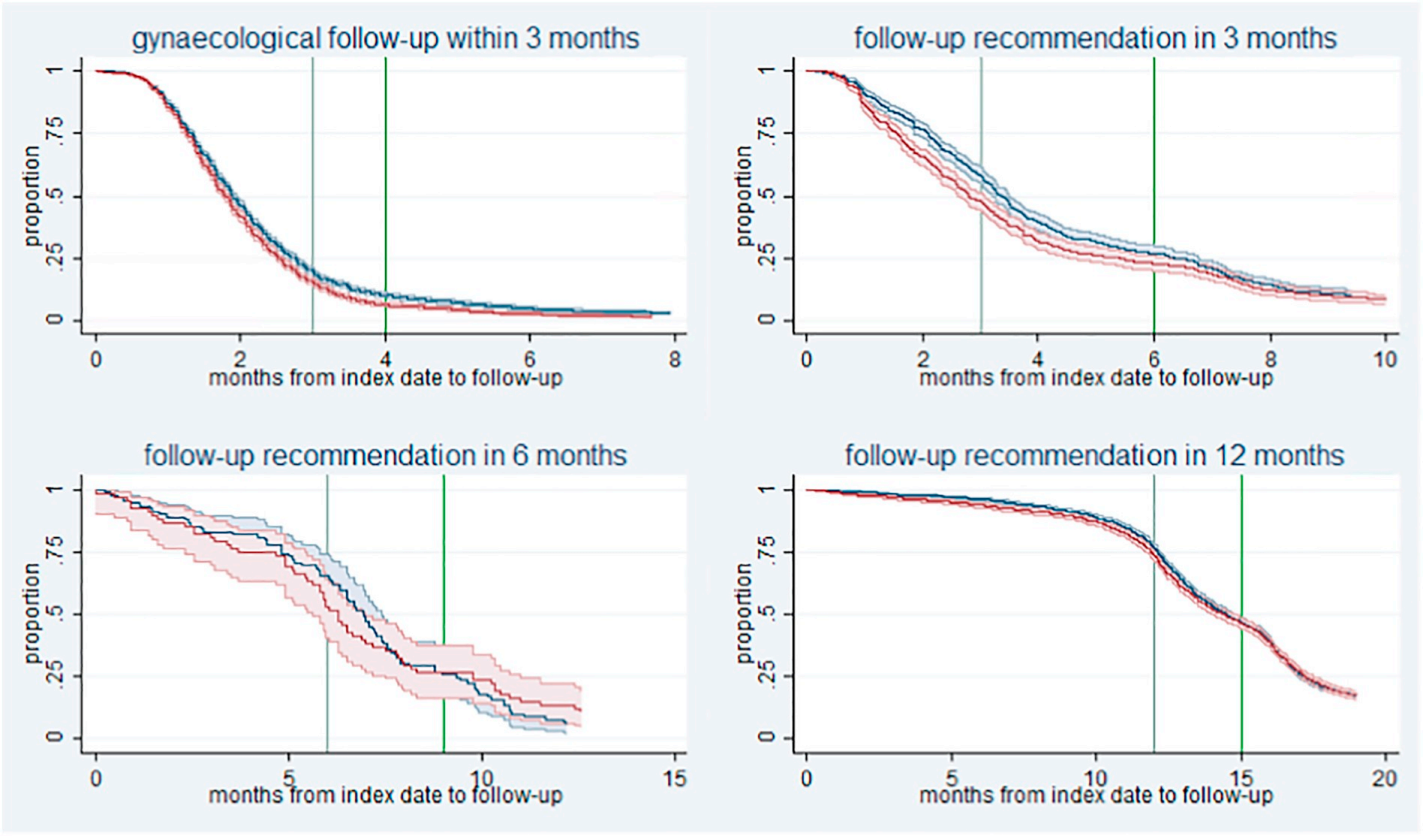
Proportions of women without follow-up among women receiving usual care (control (blue)) or direct notification by letter (intervention (red)), stratified by follow-up recommendation^1^. Observation of effect stopped 4 months after the GP reminder was generated. ^1^Time point 0 represents the date that the cervical cytology was obtained. The first vertical line is the time point for recommended timely follow-up. The second vertical line is the time point at which a reminder is generated by the screening programme and sent to the GP, if the woman has not had follow-up. A GP reminder is generated 4 months after the initial cervical cytology if follow-up is recommended within 3 months, and after 6, 9, or 15 months, if follow-up is recommended in 3, 6, or 12 months, respectively.

Time from cervical cytology requisition to follow-up is displayed in Fig. 2. In these plots, the observation was prolonged to visualise the effect of the intervention in a Danish setting were GPs receive an electronic reminder in their inbox. The figure shows how differences between randomisation groups decline with time.

Table 3 presents the proportions of women without follow-up in different sociodemographic strata 1 month after the follow-up recommendation. In all sociodemographic strata, we found a (significantly or insignificantly) reduced risk of having no follow-up in the intervention group on both a relative and an absolute scale. Testing for interaction did not reveal different effects of the intervention depending on age, ethnicity or education. However, for cohabitation status, the intervention generally had a better effect among women living alone than among cohabitating women. Adjusting ethnicity, age and cohabitation for educational status and adjusting all sociodemographic groups for number of GPs per cluster only changed the estimate slightly (results not shown).

**Table 3:** The effect of direct notification of cervical cytology results to the women measured as prevalence ratio (PR) and prevalence differences (PD) of not having follow-up at different time points one month after recommended follow-up by type of sociodemographic status.

	Control	Intervention	Unadjusted PR (95% CI)	*P*-value^a^	Unadjusted PD (95% CI)	*P*-value^a^
	% without follow-up	% without follow-up				
Age group (years)						
23–34	34.1	31.5	0.92 (0.85; 1.00)		−0.03 (−0.05; 0.00)	
35–44	38.9	33.4	**0.86 (0.78; 0.95)**	0.216	**−0.06 (−0.09; −0.02)**	0.160
45–54	32.9	30.2	0.91 (0.79; 1.06)	0.874	−0.03 (−0.07; 0.02)	0.905
55–64	32.4	28.6	0.88 (0.74; 1.05)	0.630	−0.04 (−0.09; 0.01)	0.678

Ethnicity						
Danish	34.7	31.4	**0.90 (0.85; 0.96)**		**−0.03 (−0.05; −0.01)**	
Western	38.7	33.1	0.86 (0.63; 1.16)	0.730	−0.06 (−0.16; 0.05)	0.687
Non-western	39.9	30.5	0.76 (0.57; 1.03)	0.264	−0.09 (−0.19; 0.01)	0.235

Education (years)						
≤10	34.5	30.2	0.87 (0.75; 1.02)		−0.04 (−0.09; 0.01)	
>10 ≤ 15	34.6	31.0	**0.89 (0.83; 0.97)**	0.951	**−0.04 (−0.08; −0.01)**	0.951
>15	35.8	32.7	0.91 (0.83; 1.01)	0.552	−0.03 (−0.06; 0.00)	0.549

Cohabitation status						
Cohabitating	36.0	33.6	0.93 (0.87; 1.00)		−0.02 (−0.05; 0.00)	
Living alone	33.7	28.0	**0.83 (0.76; 0.91)**	**0.036**	**−0.06 (−0.08; −0.03)**	0.062

Bold characters = p-value < 0.05

a Test for interaction; showing if effect of direct notifications on a relative or absolute scale is modified by sociodemographic factors.

The ICC was estimated to be 0.010 (95% CI: 0.004 to 0.016), corresponding to a required variance inflation factor of 1.2 (with an average cluster size of 21 women).

## 4 Discussion

This study found that direct notification of cervical cytology results delivered by postal letter to women who are in need of follow-up increased the absolute proportion of women with timely follow-up by approximately four percentage points (PD: −0.04 (−0.07; −0.02)), and that it worked well in all the investigated sociodemographic groups. Still, >30% of all intervention women had received no follow-up 1 month after their recommended follow-up. The largest relative effect was found for the subgroup of women with the most severe abnormal cervical cytology results (HSIL/AIS and ASC-H), where only 6.4% of the intervention women were without follow-up 1 month after their date of recommended follow-up, compared with 10.2% of the women in the control group.

### 4.1 Strengths and limitations

The Danish National Pathology Registry and Data Bank (DPBD) register is virtually complete (Erichsen et al., 2010; Dugue et al., 2012), and selection bias is negligible. The calculated variance inflation factor was smaller than expected, which ensured a high statistical power. It was a strength that all samples were included, regardless of sample indication (screening, opportunistic or surveillance). This was done to ensure follow-up among opportunistic and surveillance samples as well, as they tend to have larger proportions of cytological abnormalities than screening samples (Sundhedsstyrelsen, 2012; Tranberg et al., 2015). Certain groups of women were excluded from the analysis, and the proportion of these groups may vary much between screening settings, and the results do not apply for these groups. The randomised controlled design with intention-to-treat analysis secured equally distributed confounders, and the cluster randomisation allowed for GPs to adjust their management in a uniform way and to avoid containment. A potential limitation was uncertainty regarding the successfulness of randomisation as randomisation baseline characteristics differed marginally, and confounding may thus have been present. Solo GP practices are more common is some areas of the CDR, and this may also partly explain why the educational status was unequally distributed among included women. In the control group, more women were highly educated. These women usually have better follow-up (Hui et al., 2014). Therefore, we adjusted for both educational status and number of GPs per cluster, but this only changed the estimates slightly. Only the intervention GPs were officially informed of the study at intervention start, but we do not consider this to have caused bias. Instead we reason that control GPs were quite familiar with the study as it raised initial debate and due to cooperation between GPs in many other ways.

### 4.2 Comparison with other studies

In 1995, Del Mar and colleagues found that letters improved the 1-year follow-up for women with abnormal cervical cytology screening results (Del Mar and Wright, 1995). To our knowledge, no other study had previously explored follow-up adherence after direct notification in cancer screening, regardless of type of screening. In the present study, we confirmed that direct notification had a positive effect on follow-up. We hypothesize that the effect is related to the advantages of letters being delivered systematically to all women with only few days of delay. Furthermore, letters may have allowed women to adopt an active role and have prepared them ahead of the consultation with the GP. This may have legitimised questions and voicing of concerns, which may in turn have minimised the risk of misunderstandings regarding test results (Lyratzopoulos et al., 2015; Kinnersley et al., 2008).

We found that the relative effect was largest for women with a recommended follow-up in 3 months or recommended gynaecological follow-up within 3 months, and smaller for women with a later follow-up (recommendation in 12 months). This may relate to a sense of urgency among the women, challenges of women and GPs planning ahead, or it may relate to women's difficulties understanding the justification of the watchful waiting approach related to the reversibility or the recurrence of abnormalities (Lee Mortensen and Adeler, 2010; Goldsmith et al., 2006; Zapka et al., 2004).

We also found that the best relative effect of direct notifications depended on the severity of the cervical cytology diagnosis. Del Mar and colleagues found a similar interaction (Del Mar and Wright, 1995). For instance, we found that among women with a recommended gynaecological follow-up within 3 months, only the women with the most severe results (HSIL/AIS or ASCH) benefitted from the intervention. To some extent this is a surprise, as all women with recommended gynaecological follow-up within 3 months had the same direct notification letter phrased as ‘not normal’, regardless of the severity of their cervical cytology results. Moreover, women fear cancer to the same extent regardless of the severity of their results, and do not know the technical jargon of milder and more severe diagnoses (Lee Mortensen and Adeler, 2010). The legal responsibility for securing follow-up in both randomisation groups may have triggered, that intervention GPs contacted women as they also did before start of study. We hypothesize that it is both likely and natural that GPs have different attention on securing follow-up depending on severity of abnormality. For instance, GPs have been found to downplay the seriousness of disease to decrease women's anxiety (Bro et al., 2008). Moreover, often women rely more on information provided by GPs than on information provided by the cervical cancer screening programme (Dieng et al., 2013). This may explain why the effect was less among less severe diagnoses, and highlights how direct notifications may interact with other contextual factors affecting choices about follow-up.

Finally, we found that the effect of the intervention was most pronounced before and just after the time of recommended follow-up, and that it diminished during the following months (Fig. 2). Possibly, this is so because Danish GPs are reminded if the women do not undergo followed-up, which acts as a second way to secure follow-up.

Sociodemographic inequality is present at several levels in cervical cancer prevention (Slattelid Schreiber et al., 2015; von Wagner et al., 2011; Zapka et al., 2014). For instance, younger single women with lower educational levels and women from ethnic minority groups have a higher risk of not undergoing follow-up (Eggleston et al., 2007; Khanna and Phillips, 2001). It was a concern in the present study that direct notifications by letter might increase this inequality; for instance because these groups tend to report that it is difficult to understand written health information (Bo et al., 2014). However, our results indicate that the intervention did not increase social inequality. On the contrary, the intervention worked significantly better among single women than among cohabitating women. All notifications were written in Danish lay language, and it was encouraging that the intervention also benefitted non-Western Danes.

### 4.3 Implications

Even though women may prefer direct notifications of results (Philips et al., 2002; Simon et al., 2010), this approach is not commonly used in cervical cancer screening (Arbyn et al., 2008; Anttila et al., 2009; Elfstrom et al., 2015; Linos and Riza, 2000). On the contrary, use of direct notifications is debated internationally due to the risk of interference by the relationship between the woman and the GP (Solomon et al., 2002). Some studies have found that communication of alerts to more than one recipient double the risk of not receiving timely follow-up, maybe because it is less clear who is responsible (Singh et al., 2009). The conclusion of the present study may contribute to the development of future cervical cancer screening guidelines.

In general the intervention had limited effect, and this highlights importance of further strategies to improve follow-up: It is possible that refined phrasing in letters will improve the effectiveness of direct notifications. Especially, it may be relevant for women with a recommendation for follow-up in 12 months and a letter phrased as ‘normal’, as they had no effect of direct notifications. This group has often had a previous abnormal result, and follow-up is important as recurrence of abnormalities is common (Sundhedsstyrelsen, 2012). Letters may also be refined for subgroups of women, for example by translating letters into minority languages or including a motivational brochure when sending the letter (Simon et al., 2010). It may also be possible to combine direct notifications with structured telephone counselling to counter psychological barriers (Miller et al., 2013), or reminding the women, just like GPs are reminded (Khanna and Phillips, 2001). This could be important as women perceive multiple barriers for attending follow-up (Eggleston et al., 2007).

Women's acceptance of notification letters must also be acknowledged as an important consideration before implementation of letters. In our study, all women could decline receiving a direct notification by contacting the Department of Public Health Programs (through their GP, if preferred), but few women used this opportunity. In the study by Del Mar, <50% of the women accepted to receive the results by direct notification letter, but the GPs in their study needed to record the woman's current address during the GP consultation, which may have been a barrier (Del Mar and Wright, 1995). Thus, different scenarios for acceptance of direct notifications will most likely influence the effect of follow-up and the sociodemographic variation.

## 5 Conclusion

Direct notification of cervical cytologies may be a simple way to improve women's adherence to follow-up in a cervical cancer screening programme in general and adherence of women with a need for earlier follow-up in particular. Timely follow-up increased by four percentage points when the women received a direct notification of their cytology result after their screening. All groups of women benefitted from the intervention regardless of age, educational level, ethnicity and cohabitation status. Despite the positive effect of direct notification on timely follow-up, 30% of the women were still without follow-up 1 month after the date of follow-up recommendation. Further interventions are thus needed.

The following are the supplementary data related to this article.

Appendix A The Danish national screening programme, Diagnostic classifications systems and the SNOMED-code algorithm. Appendix A

Appendix B Distribution of cytology diagnosis by follow-up recommendation (*n*, %). Appendix B
