# Supplementary material for: Direct notification of cervical cytology results to women improves follow-up in cervical cancer screening - A cluster-randomised trial
Source: Prev Med Rep. 2018 Nov 23;13:118–25. doi: 10.1016/j.pmedr.2018.11.015 (PMC6296289; doi:10.1016/j.pmedr.2018.11.015)
Supplement: Appendix A — The Danish national screening programme, Diagnostic classifications systems and the SNOMED-code algorithm. [file mmc1.docx]

## Appendix A

1. Danish National Screening Programme and follow-up recommendations (24)

| Screening (cervical cytology test): 23-60 years of age | | | | Screening (HPV test): 60-64 years of age | | | | After cone biopsy |
| --- | --- | --- | --- | --- | --- | --- | --- | --- |
| Normal | ASCUS/LSIL | ASCH/AGC HSIL/AIS | Inadequate | HPV neg. | HPV pos.  (not 16 or 18) | HPV pos.  (16 or 18) | Inadequate  HPV test | Cytology morphology and HPV tests are performed. Further earlier histology results  determine follow-up recommendations |
|  | If Ascus, triage with HPV test |  |  |  | Triage with cytology morphology test |  |  |  |
| Follow regular screening interval | Follow regular screening interval or follow-up before 3 (gynaecologist), in 6 or 12 months | Follow-up  before 3 months  (gynaecologist) | Follow-up  before 3 months (gynaecologist)or  in 3 months | Screening programme ends | Follow-up before 3 (gynaecologist) or in 3 or 12 months | Follow-up before 3 months (gynaecologist) | Follow-up before 3 (gynaecologist) or in 3 months | Follow regular screening interval or  follow-up before 3 (gynaecologist), in 6 or 12 months |

1. Diagnostic classification systems (24)

| Histological classification of diagnosis | | | | | | | | | |
| --- | --- | --- | --- | --- | --- | --- | --- | --- | --- |
| CIN | Normal | | Atypia | CIN I | CIN II | CIN III | | Carcinoma | Inadequate |
| Cytological classification of diagnosis | | | | | | | | | |
| Modified WHO | Normal | | Atypia | HPV + Mild dysplasia | Moderate dysplasia | Severe dysplasia | Carcinoma in situ | Carcinoma | Inadequate |
| Bethesda 2001 | Normal *with* organisms or non-neoplastic findings | Normal *without* organisms or non-neoplastic findings | ASCUS/ASCH/AGC | LSIL/HPV | HSIL/AIS | | | Adeno Carcinoma /SCC | Unsatisfactory for evaluation |

1. SNOMED-coding algorithm used to generate result notifications for cervical cytology samples**^1^** (24,32)

| Diagnose | | | | | | | | | | |
| --- | --- | --- | --- | --- | --- | --- | --- | --- | --- | --- |
| Morphology: HSIL/AIS/ASCH/AGC/ Adeno Carcinoma /SCC | Morphology:  ASCUS/  LSIL | Morphology:  ASCUS/  LSIL | HPV-pos.  Morphology: normal **OR** unknown | Morphology:  Unsatisfactory for evaluation | Morphology:  Normal **with** organisms or non-neoplastic findings | Morphology:  Normal | | HPV-neg.  Morphology:  unknown | | Unknown diagnose^2^ |
| SNOMED codes to determine diagnose | | | | | | | | | | |
| M67010 M67020 M67017 M81403 M80013 M80103 M69760 M74B09 M74BK9 M74C09 M80102 M81402 M807A2 M80703 M83803 | M67016  M67014  M69700 | M67016 M67014 M69700 | FY5006 FY5009 FY5010 Æ33416 Æ33418 Æ33431 Æ33433 Æ33435 Æ33439 Æ33445 Æ33451 Æ33452 Æ33456 Æ33458 Æ33459 Æ33466 Æ33468 | M09010 M09070 M09100 M09150 M0901H | M00120 M00121 M00122 AND M02561 M02562 M69810 ÆAA016 MÆ0024 Æ44330 Æ32100 Æ32110 | M00120 M00121 M00122 | | FY5005 FY5001 | |  |
| SNOMED codes to determine follow-up | | | | | | | | | | |
| Regardless of  follow-up  SNOMED  codes | Gynaecological follow-up within 3 months (contact GP): ÆAAX15 ÆAA015  **OR** In 3 months: ÆAA001 ÆAA0Y0 ÆAA021 ÆAA0Y1  **OR** In 6 months: ÆAA004 ÆAA0Y2 ÆAAXY2 ÆAA005 ÆAA006;  **OR** In 12 months: ÆAA0Y3 ÆAA018 ÆAA008, ÆAA009 ÆAA00E ÆAA00A | No follow-up SNOMED  code is present | Regardless of  follow-up  SNOMED  codes | Regardless of  follow-up  SNOMED  codes | Regardless of  follow-up  SNOMED  codes | No follow-up SNOMED  code is present | Gynaecological follow-up within 3 months (contact GP): ÆAAX15 ÆAA015  **OR**  In 3 months: ÆAA001 ÆAA0Y0 ÆAA021 ÆAA0Y1 **OR** In 6 months: ÆAA004 ÆAA0Y2 ÆAAXY2 ÆAA005 ÆAA006  **OR** In 12 months: ÆAA0Y3 ÆAA018 ÆAA008, ÆAA009 ÆAA00E ÆAA00A | No follow-up SNOMED  code is  present | Gynaecological follow-up within 3 months (contact GP): ÆAAX15 ÆAA015  **OR** In 3 months: ÆAA001 ÆAA0Y0 ÆAA021 ÆAA0Y1 **OR** In 6 months: ÆAA004 ÆAA0Y2 ÆAAXY2 ÆAA005 ÆAA006  **OR** In 12 months: ÆAA0Y3 ÆAA018 ÆAA008, ÆAA009 ÆAA00E ÆAA00A | Regardless  of follow-up SNOMED codes |
| Wording in the direct notification by postal letter | | | | | | | | | | |
| Your test is  not normal.  Contact your GP for follow-up | Your test is  not normal  AND  Contact your GP for follow-up  **OR** Contact your GP and have a new test in 3, 6, **OR** 12 months | Contact your GP to have your  test result | The test shows  high risk HPV.  Contact your GP  for follow-up | The test was insufficient.  Contact  your GP and have  a new test | Contact  your GP to  have your  test result | Your test is normal  Await new screening invitation or screening ends **^4^** | Your test is  normal  AND  Contact your GP for follow-up  OR Contact your GP and have a new test in 3, 6, OR 12 months ^3^ | Your test is  normal  Await new screening  Invitation or screening  ends **^4^** | Your test is  normal  AND  Contact your GP for follow-up  **OR** Contact your GP and have a new test in 3, 6, **OR** 12 months **^3^** | Contact  your GP to have your  test result |

**^1^** The algorithm was applied to topography codes T8X310 and T8X320. To generate a letter, specific SNOMED codes had to be present, and others were not allowed to be present (for example, it was not possible to use a normal SNOMED code with an abnormal code). In few cases, the woman was not contacted by letter (unknown address, changed diagnosis or follow-up recommendation after dispatch of the results to GPs). In these cases, the GP was notified by postal letter and asked to convey the test results to the women.

**^2^** The “unknown diagnosis letter” was used if other categories could not be fulfilled (i.e. ambiguous SNOMED coding, imprecise follow-up recommendation, or cytology samples with topography codes T8X210, T8X311, T8X312, or T8X330). These women were in the letter informed to contact the GP to have the result as were women with a normal result with “organisms or non-neoplastic findings” (e.g. herpes).

**^3^** Women with a normal result may be recommended follow-up if they have had an earlier abnormal result, as part of a surveillance programme.

**^4^** Women with a normal test result who could follow regular screening intervals where not included in the study.
