# Supplementary material for: Direct notification of cervical cytology results to women improves follow-up in cervical cancer screening - A cluster-randomised trial
Source: Prev Med Rep. 2018 Nov 23;13:118–25. doi: 10.1016/j.pmedr.2018.11.015 (PMC6296289; doi:10.1016/j.pmedr.2018.11.015)
Supplement: Appendix B — Distribution of cytology diagnosis by follow-up recommendation (n, %). [file mmc2.docx]

**Appendix B**

Distribution of cytology diagnosis by follow-up recommendation (n, %)

|  | **Recommended follow-up** | | | |  |
| --- | --- | --- | --- | --- | --- |
| **Diagnosis** | Within 3 months^1^ | In 3 months^2^ | In 6 months^3^ | In 12 months^4^ | Total |
| Normal | 20 (0.4) | 3 (0.2) | 77 (49.0) | 2,890 (63.1) | 2,990 (25.3) |
| Inadequate | 39 (0.7) | 1,589 (99.5) | 15 (9.6) | 1 (0.0) | 1,664 (13.9) |
| Abnormal |  |  |  |  |  |
| Ascus | 263 (4.8) | 4 (0.3) | 23 (14.7) | 13 (0.3) | 303 (2.6) |
| Ascus HPV neg. | 103 (1.9) | 0 | 5 (3.2) | 722 (15.8) | 830 (7.0) |
| Ascus HPV pos. | 1,009 (18.4) | 0 | 2 (1.3) | 0 | 1,011 (8.5) |
| AGC | 151 (2.8) | 0 | 1 (0.6) | 0 | 152 (1.3) |
| AGC HPV neg. | 2 (0.0) | 0 | 0 | 0 | 2 (0.0) |
| AGC HPV pos. | 2 (0.0) | 0 | 0 | 0 | 2 (0.0) |
| ASCH | 1,269 (23.1) | 0 | 8 (5.1) | 0 | 1,277 (10.8) |
| ASCH HPV neg. | 17 (0.3) | 0 | 0 | 0 | 17 (0.1) |
| ASCH HPV pos. | 12 (0.2) | 0 | 0 | 0 | 12 (0.1) |
| HPV pos. | 307 (5.6) | 0 | 4 (2.6) | 77 (1.8) | 388 (3.3) |
| LSIL | 343 (6.2) | 1 (0.1) | 12 (7.6) | 28 (0.6) | 384 (3.3) |
| LSIL HPV neg. | 53 (1.0) | 0 | 2 (1.3) | 852 (18.6) | 907 (7.7) |
| LSIL HPV pos. | 617 (11.2) | 0 | 1 (0.6) | 1 (0.0) | 619 (5.2) |
| HSIL/AIS | 1,252 (22.8) | 0 | 7 (4.5) | 0 | 1,259 (10.6) |
| HSIL/AIS HPV neg. | 2 (0.0) | 0 | 0 | 0 | 2 (0.0) |
| HSIL/AIS HPV pos. | 13 (0.2) | 0 | 0 | 0 | 13 (0.1) |
| Cancer | 21 (0.4) | 0 | 0 | 0 | 21 (0.2) |
| Total | 5,495 (100.0) | 1,597 (100.0) | 157 (100.0) | 4,584 (100.0) | 11,833 (100.0) |
